# The effect of a single dose of escitalopram on sensorimotor networks

**DOI:** 10.1002/brb3.975

**Published:** 2018-04-20

**Authors:** Christian Weisstanner, Georg Kägi, Werner Krammer, Chin B. Eap, Roland Wiest, John H. Missimer, Bruno J. Weder

**Affiliations:** ^1^ Support Center for Advanced Imaging (SCAN) Department of Diagnostic and Interventional Neuroradiology, Inselspital Bern University Hospital Bern Switzerland; ^2^ Department of Neurology Kantonsspital St. Gallen St. Gallen Switzerland; ^3^ Unit of Pharmacogenetics and Clinical Psychopharmacology Department of Psychiatry Center for Psychiatric Neuroscience Lausanne University Hospital Prilly Switzerland; ^4^ School of Pharmaceutical Sciences University of Geneva and University of Lausanne Geneva Switzerland; ^5^ Laboratory of Biomolecular Research Paul Scherrer Institute (PSI) Villigen Switzerland

**Keywords:** dynamic causal modeling, escitalopram, fMRI, motor control, principal component analysis, sensorimotor networks

## Abstract

**Introduction:**

Serving as a pilot study of poststroke pharmacotherapy, the present investigation was intended to establish the effect of a single dose of escitalopram on motor task performance in normal volunteers.

**Methods:**

Ten healthy volunteers of median age 63 years including four females performed a well‐studied tactile manipulation task in two fMRI sessions using a double‐blind cross‐over design. The sessions began approximately three hours after ingestion of 20 mg escitalopram or placebo presented in pseudorandom order. The fMRI image sequences were submitted to principal component analysis (PCA).

**Results:**

Based on volume correlations of task‐related principal components with the mean component images derived in our previous study, we established the reproducibility of two networks of sensorimotor activity proposed there. The network reflecting motor control (cerebral pattern I) appeared invariably in placebo and verum conditions. In contrast, the other network, attributed to diminished motor control due to distracting mental processing (cerebral pattern II), emerged less regularly and exhibited more variability. Second‐level PCAs of both conditions confirmed the findings of the initial analysis. Specifically, it validated the dominant and invariable expression of cerebral pattern I after application of a single dose of escitalopram. Dynamic causal modeling confirmed enhanced motor output as a result of a significantly increased connectivity between primary motor cortex and dorsal premotor cortex.

**Conclusion:**

This pilot study suggests the promise of stimulation by a specific serotonin reuptake inhibitor in regard to recovery and preservation of motor control after stroke.

## 1 INTRODUCTION

An expert panel dealing with poststroke pharmacotherapy focused a few years ago on drugs that might facilitate motor recovery (Chollet et al., 2014). Relying on current data serotonergic agents has been appraised as interesting substances to promote recovery during the first 3 months after stroke. However, the experts deplored a lack of understanding the mechanisms of pharmacotherapy poststroke on a systemic level, that is the mechanisms of the drugs on brain network dysfunction. A special challenge is understanding recovery in animals after lesion experiments and translation to the human condition. Possibly significant aspects include emergence of novel cortical rhythms and their association with axonal sprouting, flexible adaptation of single neurons to stimuli, and, importantly, long‐term potentiation (LTP) of synapses (Carmichael, 2012; Carmichael & Chesselet, 2002; Winship & Murphy, 2008).

The ubiquity of 5‐hydroxytryptamine, 5‐HT (i.e., serotonin), receptors in the human brain has been established, and their varying distributions were quantified by high‐resolution neuroimaging data consisting of positron emission tomography (PET) and magnetic resonance (MR) imaging scans (Beliveau et al., 2016). However, animal experiments in cats show that the majority of brain 5‐HT receptor neurons lie in the brainstem raphe nuclei, of which the anterior two nuclei supply most of the 5‐HT to the forebrain, while the posterior nuclei supply the pyramidal neurons in the spinal cord (Jacobs & Fornal, 1997). Jacobs, Martin‐Cora, & Fornal (2002) found an invariable tonic motor output in cats after application of a single dose of a serotonergic agent at this site, an effect that could also be elicited by internal release of serotonin in the course of awakening and directed motor control (Jacobs & Fornal, 1995). A model of an immediate direct effect of serotonergic agents on postsynapses, reflecting an early phase of LTP, has been established on a molecular level; it depends on intracellular calcium influx and activation of calmodulin‐dependent kinase II, tyrosine kinase fyn, and protein kinase sensitizing non‐NMDA receptors to the interaction of glutamate (Kandel, 2000).

In the context of stroke recovery, a large randomized, double‐blind, placebo‐controlled trial (FLAME trial) involving 118 patients poststroke evinced significant motor improvement after administration of the selective serotonin reuptake inhibitor (SSRI) fluoxetine for 3 months (Chollet et al., 2011). Pariente et al. (2001) found enhanced motor output in subcortical stroke patients after application of a single dose of fluoxetine; Loubinoux et al. (2002) observed an increased motor output in a paced motor task in normal volunteers upon administration of paroxetine. Escitalopram is a highly selective SSRI that has minimal effects on other neurotransmitter systems, for example norepinephrine or dopamine (Kasper et al., 2009). Its property indicates that a single dose of escitalopram, as administered in this study, is most suitable for evaluating the effect of serotonergic motor stimulation on a systemic level.

A well‐studied tactile manipulation task investigated using fMRI provides a suitable test of motor task performance. In a principal component analysis of the task performed by seven normal volunteers, we have distinguished in this previous study two distinct neural networks underlying tactile object manipulation (Kagi, Missimer, Abela, Seitz, & Weder, 2010). A hierarchical classification of the principal components computed for each subject yielded two mean component images representing the networks. As expected during the grasping of objects, both mean component images displayed the primary motor and somatosensory cortex. The first mean component image, that is the component most strongly and invariably expressed, suggested directed motor control. Dynamic causal modeling (DCM) provided the opportunity to assess the directed effective connectivity among the core nodes of the distributed network represented by the component, that is the primary motor cortex (M1), dorsal premotor cortex, and supplementary motor area (SMA) (Daunizeau, David, & Stephan, 2011; Friston, Harrison, & Penny, 2003; Friston, Li, Daunizeau, & Stephan, 2011; Stephan et al., 2010). The second component image, expressed less strongly and frequently than the first, indicated diminished motor control due to interference of stimulus‐independent mental processes (e.g., attentional and orienting stimuli) during the task (Jacobs & Fornal, 1995). The pilot study reported here enlisted a cohort of ten normal volunteers suited for a study of poststroke recovery. We hypothesized that the network of directed motor control will be preferentially expressed after administration of a single dose of escitalopram (verum condition), whereas the second network of diminished motor control would be expressed predominantly in the same volunteers after administration of a placebo (placebo condition). A prior condition for the comparison was the reproducibility of the two previously proposed networks of sensorimotor activity in a cohort of older volunteers.

## 2 SUBJECTS AND METHODS

### 2.1 Subjects

Ten healthy volunteers were included, six were males and four were females; the age of the subjects ranged between 50 and 74 years with a mean age of 62. Eight subjects were right‐handed according to the Edinburgh handedness inventory (Laterality quotient, LQ, 100). Two subjects were ambidextrous, one with a right‐hand preference and a LQ of 30, and one with a left‐hand preference and a LQ of −60 (Oldfield, 1971). None of them presented neurological or psychological disorders at the time of the study; furthermore, there was no intake of medication which might interfere with escitalopram.

In order to assess the effects of age in comparing these subjects with those of our previous study (Kagi et al., 2010), ten age‐matched subjects were selected from a follow‐up study. The cohort consisted of four males and six females of mean age 65.5 years ranging between 54 and 75 years. With the exception of an ambidextrous man, all were right‐handed with an average laterality quotient of 84.4. None of them presented neurological or psychological disorders at the time of the study.

Prior to inclusion in the study, written consent was obtained from all subjects according to the Declaration of Helsinki. The study was approved by the ethics committees of both research centers that carried out the acquisitions [Ethikkommission Ostschweiz (EKOS), Kantonsspital St. Gallen, 9007 St.Gallen and Kantonale Ethikkommission Bern (KEK), 3010 Bern, Switzerland].

### 2.2 Methods

#### 2.2.1 Study design

To measure the effect of a single dose of 20 mg escitalopram on the performance of a sensorimotor task, each subject performed the task in two fMRI sessions according to a double‐blinded cross‐over design. The sessions followed 3–4 hr after ingestion of either a placebo or 20 mg escitalopram. The order of the sessions was pseudorandomized over subjects; five received placebo before the first session and five escitalopram. The median time between the two sessions was 30.5 (range 18–63) days. Subjects of the age‐matched control group ingested nothing prior to their single fMRI session.

#### 2.2.2 Measurement of escitalopram serum level

A blood drawing was performed before each session (i.e., 3–4 hr after ingestion of escitalopram). Serum concentrations of escitalopram were measured by liquid chromatography/mass spectrometry as previously described (Ansermot, Brawand‐Amey, & Eap, 2012). *CYP2C19* genotypes (analysis of *2 (rs4244285), *3 (rs4986893), and *17 (rs12248560) alleles) and *ABCB1* genotypes (analysis of G2677T (rs1045642) and C3435T (rs2032582) alleles) were determined as previously described (Crettol et al., 2006).

#### 2.2.3 Stimulation paradigm

The task consisted of sequential manipulation of nonmagnetic hard aluminum cubes with equal mass (32.5 g) and volume (11.5 cm^3^) presented to the subjects’ right hand by an investigator standing next to the scanner. Subjects were instructed to tactually manipulate the spheres in their right hand during the 6 s presentation time with a thumb frequency of about 1 Hz. No further specific instructions on hand movements were given, and no explicit discrimination of the spheres was required as the objects were different neither in shape nor size. Subjects lay supine inside the scanner with their heads immobilized and their eyes closed. The investigator received the signal to present and remove the spheres via light projection of the instruction from outside the scanner room. Each cube was presented for 6 s, while the intervals between object presentations varied pseudorandomly between 8 and 12 s, implying repetition frequencies between 0.055 and 0.13 Hz. This ensured stochastic onsets of all conditions in relation to the image acquisition time, providing equal sensitivity in all slices of the acquired image volume. An fMRI scan consisted of 224*6 s event‐related frames during which the subject manipulated the spheres 84 times. Each subject was scanned twice during each session, yielding a total of 40 scans for the 10 subjects. To permit offline analysis of finger movements during task, the sessions were recorded by a video recorder located outside of the scanner room viewing the subjects’ hand close‐up through a window.

Except for the length of presentation and duration of the pause between presentations, the number of frames in an acquisition and the number of acquisitions, the paradigm, and its description are identical to those of the previous study (Kagi et al., 2010).

The fMRI session of the age‐matched control group consisted of three conditions, while the subjects were observing a video screen: being relaxed while visually fixating on a stationary image showing a cube in one hand (representing baseline), passive viewing of the manipulation task performed with a thumb frequency of 1 Hz, and performance of the task as observed during viewing while fixating on the stationary image again. Each condition was initiated by a visual cue. Each visual cue of 4 s, consisting of an instruction, was followed by the according main condition of 20 s (for details of the protocol, see Table S1). A training session outside of the scanner was performed immediately prior to examination in the scanner. The intention of this procedure was to assure in this elderly cohort an average frequency of thumb movement of approximately 1 Hz.

#### 2.2.4 Image acquisition

Scans were acquired using a whole‐body 3T MRI scanner (Siemens Magnetom Verio, Siemens, Erlangen, Germany). Covering the whole brain, image volumes consisted of 32 transaxial slices parallel to the bicommissural plane with a minimal resolution in plane of 3.6 × 3.6 mm and a slice thickness of 3.6 mm. The activation tasks employed an echo planar imaging–blood oxygen level‐dependent (EPI–BOLD) sequence with time of acquisition (TA) of 456 ms, time of repetition (TR) of 2000 ms, time of echo (TE) of 30 ms, flip angle of 90°, and phase encoding direction from anterior to posterior. No specialized shimming procedure was used. Of the 227 volumes acquired in each scan, the first three were discarded from the analysis. For each subject, an anatomical T1‐weighted image with high resolution consisting of 176 sagittal slices and 1.0 × 1.0 mm in‐plane resolution, slice thickness of 1.0 mm, was also acquired with a TA of 353 ms, TR of 2530 ms, TE of 2.2 ms, flip angle of 9°, phase encoding direction from anterior to posterior, and no fat suppression.

#### 2.2.5 Data analysis

Except for the version of MATLAB [The MathWorks, Inc., Natick, MA], the number of acquisitions, frames and relevant voxels, image preprocessing, and PCA and their description are identical to those of the previous study (Kagi et al., 2010).

#### 2.2.6 Image preprocessing

Image data were analyzed using SPM 12 (Wellcome Trust Centre for Neuroimaging, London, UK) implemented in MATLAB^®^ (R2012a). Functional images of each acquisition were slice time‐corrected, realigned to the sixteenth volume of the functional imaging series, and coregistered to the subjects’ anatomical T1‐weighted mean images. The images were then normalized into the standard space defined by the Montreal Neurological Institute (MNI) template and smoothed using a Gaussian kernel with full width at half maximum of 8 × 8 × 8 mm. The dimensions of the resampled images were 79 × 95 × 68 voxels and the voxel sizes 2 × 2 × 2 mm^3^.

#### 2.2.7 Principal component analysis

The preprocessed fMRI time series served as input to in‐house software written in MATLAB [The MathWorks, Inc., Natick, MA, USA] based on the algorithm described by Alexander and Moeller (Alexander & Moeller, 1994). Extracerebral voxels were excluded from the analysis using a mask derived from the gray matter component yielded by segmentation of the anatomical image volume into gray matter, white matter, and cerebrospinal fluid. Calculation of residual matrices for each of the 40 acquisitions followed. From matrices whose rows corresponded to the 224 time frames of a scan and columns to the 151411 relevant voxels in a single image volume were subtracted from each element (i) the mean of voxel values of its column and (ii) the mean of voxel values of its row and (iii) added to each element the grand mean of all voxel values in the original matrices. The row, column, and grand means of the resulting residual matrices vanish. Using the singular value decomposition implemented in MATLAB, each residual matrix was then decomposed into 224 components. Each component consisted of an image volume, that is eigenimage, a temporal expression coefficient, that is eigenvariate, and an eigenvalue: The squared eigenvalue is proportional to the fraction of variance described by each component; the temporal expression coefficients describe the amount that each scan contributes to the component; and the component image displays the degree to which the voxels covary in the component. The temporal expression coefficients and voxel values of a principal component are orthonormal and range between −1 and 1; the orthogonality reflects the lack of statistical correlation among the principal components.

The PCAs of the age‐matched control group differed only in the fewer number of acquisitions, namely 10, and of the time frames analyzed, namely 84.

#### 2.2.8 Selection criteria for task‐related principal components

Correlation between the temporal expression coefficients and the hemodynamic response (ecc) determined the task‐related principal components. The 224 temporal expression coefficients derived from each of the acquisitions imply the same number of correlation coefficients. Achieving a significance of *p *< .01 for that number of multiple comparisons requires *p *< .01/222 = 0.000045 according to Bonferroni correction. This probability corresponds to a correlation coefficient of 0.269 (two‐tailed) for 222 degrees of freedom. Thus, correlation coefficients exceeding 0.269 were assumed to be task‐related.

Similarly, for the age‐matched control group, achieving a significance of *p *< .01 for 84 temporal coefficients required that correlation coefficients exceed 0.407 to be considered task‐related.

#### 2.2.9 Classification of task‐related principal component images

In order to classify the task‐related PCs, volume correlation coefficients (vccs) were calculated between image volumes. The two mean component images, MCI_K1 and MCI_K2, determined in the previous study (Kagi et al., 2010) and the PC image volume of the present study with the maximum ecc served as reference volumes for correlation with each of the task‐related PCs. The third reference volume mentioned above served only as a control. As the number of relevant voxels and multiple comparisons were similar in number to our previous analysis (Kagi et al., 2010), the significance threshold was assumed to be the same. The theoretical t‐distribution deduced in that analysis, consistent with 1660 degrees of freedom, required that the vccs exceed 0.09 in magnitude to achieve a significance threshold of *p *< .05 corrected for 240 multiple comparisons. However, simulated distributions derived from noise‐like PC image volumes exhibited four outliers between 0.14 and 0.18, suggesting the more conservative threshold of 0.14 and implying a significance threshold of *p *< .04 corrected for multiple comparisons. The theoretical distribution implies for this threshold *p *< 3*10^−6^ corrected for multiple comparisons.

In order to validate the activation patterns found by Kagi et al. (2010) and to study their modification due to escitalopram, vccs were employed to calculate four mean component images corresponding to the mean component images MCI_K1 and MCI_K2 for placebo and verum conditions: MCI_V1, MCI_P1, MCI_V2, and MCI_P2, where V denotes verum and P placebo while the suffix 1 indicates mean images related to MCI_K1 and 2 those related to MCI_K2. Excluded from the calculation were those task‐related PCs for which the vcc did not exceed the threshold, the vccs with the two reference component images were comparable, or another task‐related PC of the same condition and acquisition yielded a greater vcc. A fourth reason for exclusion was inconsistency between vccs of the first and second acquisitions in a subject.

In order to preclude a classification bias due to reliance on the activation patterns of Kagi et al. (2010), second‐level PCAs of the selected principal components of placebo and verum, respectively, were performed. Computed with the same PCA algorithm used in selection, these two PCAs offer a blind data‐driven classification in which the classes are orthogonal, that is, statistically uncorrelated, in contrast to the hierarchical analysis based on volume correlations used in the previous study.

For the age‐matched control group, a second‐level PCA of the 840 principal component images was also performed.

#### 2.2.10 Statistical analysis of expression and image volume correlation coefficients

The Mann–Whitney U test was applied to distinguish differences in eccs between MCI_V1 and MCI_P1 and between MCI_V2 and MCI_P2. The vccs of the image volumes constituting the condition images MCI_V1 and MCI_P1 with the mean condition image MCI_K1 were compared with an unpaired, two‐tailed t test, as were the vccs of MCI_V2 and MCI_P2 with the mean condition image MCI_K2. The Fisher transformation was applied to evaluate differences between vccs of MCI_V1 and MCI_P1 with MCI_K1 and of MCI_V2 and MCI_P2 with MCI_K2.

Analyzing the relation between eccs and vccs proved also to be fruitful. In the case of the image volumes composing MCI_V2 and MCI_P2, the Mahalanobis distance (Mahalanobis, 1936) served as a useful tool.

#### 2.2.11 Salient cerebral regions of task‐related PCs

To determine the prominent regions of a task‐related PC, thresholds were applied to the distribution of voxel values within a PC image volume; only those voxels for which the voxel values lay in the first or ninety‐ninth percentile of voxel values were considered salient. In addition, only clusters of at least 32 voxels satisfying the threshold were analyzed. These clusters were localized using the probabilistic cytoarchitectonic maps provided by SPM anatomy toolbox (Eickhoff et al., 2007).

#### 2.2.12 Connectivity analysis

Dynamic causal modeling, performed with DCM12 as implemented in SPM12, focused on the three core regions of the sensorimotor network within the left hemisphere: M1, PMC, and SMA. The three regions were represented by ROIs delineated on the subject's T1‐weighted mean image normalized to MNI space. Anatomical landmarks determined the positions of the ROIs: The hand knob located the M1 with PMC rostral to it and the intermediate part of Brodmann area 6 located the SMA. Spherical volumes of interest (VOI) of 8 mm radius described the ROIs. VOIs were centered on the highest suprathreshold voxel within a ROI; the center coordinates are listed in Table S2. For each acquisition, a first‐level analysis using the general linear model of the preprocessed spatially normalized functional images used for PCA yielded brain activation maps corresponding to the task condition “rest < move” (*p *< .05, corrected for FWE). VOI analysis corresponding to the physiological states “rest” and “move” provided input for the DCM. Based on a previous study (Friston et al., 2011), only endogenous connections were assumed between VOIs. There resulted six possible models for each condition: verum or placebo. After estimation of the endogenous coupling parameters for all models and each subject's acquisitions, the random effects Bayesian model selection algorithm implemented in DCM12 was used to identify the best‐fitting model. As the favored model in both conditions was the same, coupling parameters were compared using Mann–Whitney U test.

## 3 RESULTS

### 3.1 Analysis of finger movements

Subjects performed mainly rolls and dynamic digital movements with thumb, index, and middle fingers, a movement pattern consistent with earlier observations (Kagi et al., 2010; Seitz, Roland, Bohm, Greitz, & Stone‐Elander, 1991). The subjects performed the thumb movements preferentially along a translational axis toward the long finger and back. Frequency of the thumb, the most active finger in this type of exploratory action (Seitz et al., 1991), was assessed during dynamic digital movements by analyzing the video recordings and determining 10 representative sequences of object manipulations during an event of 6 s. As shown in Table 1, the frequency was 1.11 ± 0.22 Hz. (mean ± SD) for the placebo condition and 1.14 ± 0.27 Hz. for the verum condition, verifying that the subjects performed equally well in both conditions (*p *< .54). In the age‐matched control group, movement frequency of the thumb was 1.02 ± 0.09 Hz.

**Table 1:** Blood sampling, acquisition schedule, and thumb movement

	Escitalopram plasma level 182 min [14]^b^ after ingestion		Latency between M/P^a^ intake until		Task performance
			Begin of MRI‐task	End of MRI‐task	Thumb trajectories (frequency)
	S‐CIT (ng/ml)	S‐DCIT (ng/ml)^c^	Min	Min	Hz
Placebo group	n.a.^d^	n.a.	184.3 [16.1]	202.3 [16.1]	1.14 [0.3]
Verum group	25.1 [7.7]	2.6 [0.7]	198.6 [19.5]	216.6 [19.5]	1.11 [0.2]

a M, medication; P, placebo.

b Mean and standard deviation.

c S‐CIT, S‐enantiomer of escitalopram; S‐DCIT, metabolite of S‐CIT.

d n.a., not applicable.

### 3.2 Blood sampling and serum concentration of escitalopram

The mean escitalopram concentration was 25.1 ng/ml (SD 6.7), and mean time between intake of escitalopram and blood sampling was 181.6 min (SD 13.7). Measurement of genetic polymorphisms in the CYP2C19 and ABCB1 gene revealed three intermediate (*1/*2), five extensive (*1/*1), and two fast CYP2C19 (*1/*17) metabolizers. No significant differences were found between ABCB1 genotypes and escitalopram plasma levels or with temporal expression coefficients of components (data not shown). Table 1 lists additional details of blood sampling, acquisition schedule, and thumb movement.

### 3.3 Imaging data

**Figure 1:**
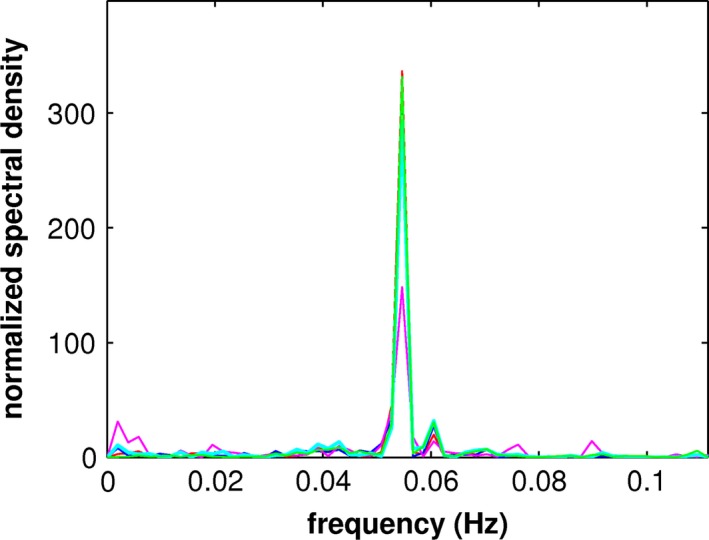
Superposition of the frequency power spectra of the mean component temporal expression coefficients for MCI_V1 and 2, MCI_P1 and 2 with the modeled hemodynamic response where red denotes MCI_V1, pink MCI_V2, blue MCI_P1, magenta MCI_P2, and green the modeled hemodynamic response

Two repetitions each of placebo and verum for each of the ten patients produced 40 acquisitions and 224 PCs for each acquisition. Computation of the eccs resulted in 83 task‐related PCs: 42 placebo and 41 verum. Except for two verum acquisitions, due to nausea as a side effect of escitalopram, every acquisition yielded at least one significant PC. All task‐related PCs fulfilled the Kaiser–Guttman criterion, which admitted the first 20 to 25 PCs of each acquisition. Of the 83 PCs, 38 placebo and 33 verum PCs yielded significant vccs with MCI_K1 or MCI_K2; the remaining 12 PCs exhibited sensorimotor activation not characterized by one of the patterns. Of the 71 PCs yielding significant vccs with MCI_K1 or MCI_K2, six produced comparable vccs with both reference image volumes. Three placebo and 12 verum PCs were redundant in the calculation of mean images related to MCI_K1 and two placebo PCs in the calculation of mean images related to MCI_K2. One placebo image volume was excluded due to an inconsistency between vccs of the first and second acquisitions. There remained 27 placebo and 20 verum PCs for computation of mean component temporal expression coefficients and image volumes: 17 placebo PCs composed MCI_P1; 15 verum PCs MCI_V1; 10 placebo PCs MCI_P2, and five verum PCs composed MCI_V2. The Fisher exact probability test showed no significant difference in sample sizes involved in comparisons of pattern I and II, respectively. The constituents of the mean temporal expression coefficients were weighted according to their eccs (see Figure S1 for graphs of mean temporal expression coefficients). Figure 1 shows the power spectra of the mean temporal expression coefficients for each of the mean components, which exhibited a prominent peak at about 0.055 Hz, the main repetition frequency of the object presentation. The power spectra are completely consistent with that of the hemodynamic response.

In the direct comparison of verum and placebo conditions, the distribution of the 15 eccs associated with MCI_V1 differs significantly (*p *< .05) from the distribution of the 17 associated with MCI_P1 according to Mann–Whitney U test, whereas the distribution of the five eccs associated with MCI_V2 does not differ significantly from that of the 10 associated with MCI_P2. As to categorization an unpaired, two‐tailed t test shows no significant difference between the vccs of the 15 image volumes composing MCI_V1 with MCI_K1 and those of the 17 image volumes composing MCI_P1 with MCI_K1. The same test does show a significant difference (*p *< .01) between the vccs of the five image volumes composing MCI_V1 with MCI_K2 and those of the 10 image volumes composing MCI_P1 with MCI_K2 (see Table 2). The Fisher transformation test confirms this result; the vccs of the mean component images MCI_V1 and MCI_P1 with MCI_K1 do not differ significantly, whereas the vccs of MCI_V2 and MCI_P2 with MCI_K2 do differ at level of significance *p *< .000.

**Table 2:**
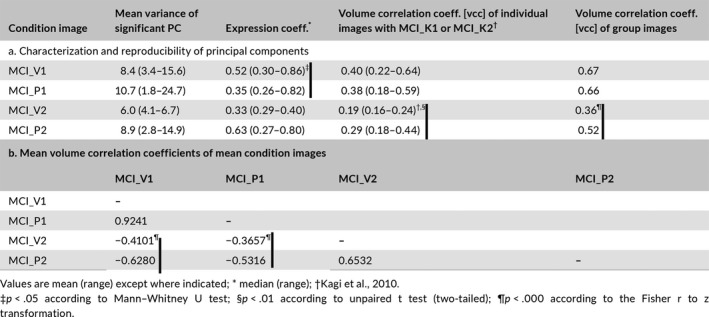
(a) Characterization and reproducibility of principal components and (b) volume correlation coefficients of mean condition images

Table 2 further shows that the vccs of MCI_V1 with MCI_P1 and of MCI_V2 and MCI_P2 are highly significant. The vccs of MCI_V2 and MCI_P2 with MCI_V1 and MCI_P2 are significant and the signs negative; that is, the second mean component images are negatively correlated with the first. The anticorrelation between MCI_V1 and MCI_V2 is less pronounced than between MCI_P1 and MCI_P2. The Fisher transformation test also shows differences at the level of significance *p *< .0000 between the vccs of MCI_V2 and MCI_P2 with MCI_V1 and MCI_P1.

**Figure 2:**
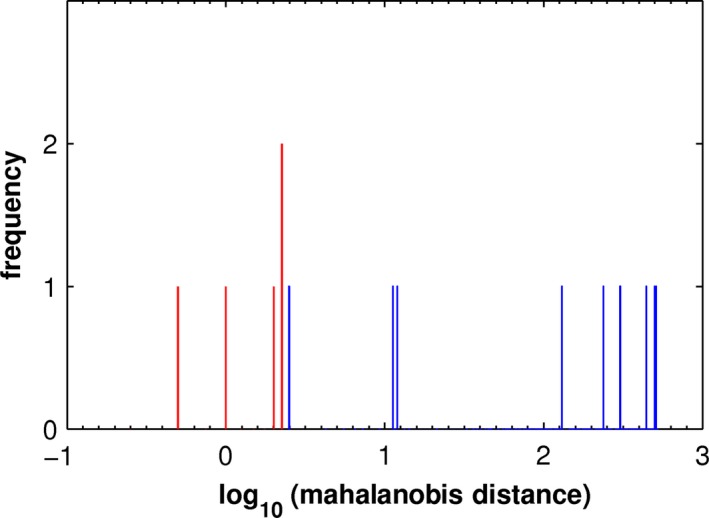
Distribution of the Mahalanobis distances in the joint distributions of eccs and vccs for cerebral pattern II: MCI_V2 (red) and MCI_P2 (blue)

The scatter plots of vccs vs. eccs for the constituents of the four mean components (see Figure S2) indicate the joint distributions for MCI_V1 and MCI_P1 show no significant correlation between vccs and eccs of their respective constituents and no significant distinction between the two distributions. In contrast, the joint distributions for MCI_V2 and MCI_P2 show trends in the correlations for both cases and suggest a distinction. Figure 2 presents an analysis of the distinction using the Mahalanobis distance (Mahalanobis, 1936). With the joint distribution of eccs and vccs constituting MCI_V2 serving as reference, the resulting distances yield a difference between the two conditions at the level of significance, *p *< .001.

**Figure 3:**
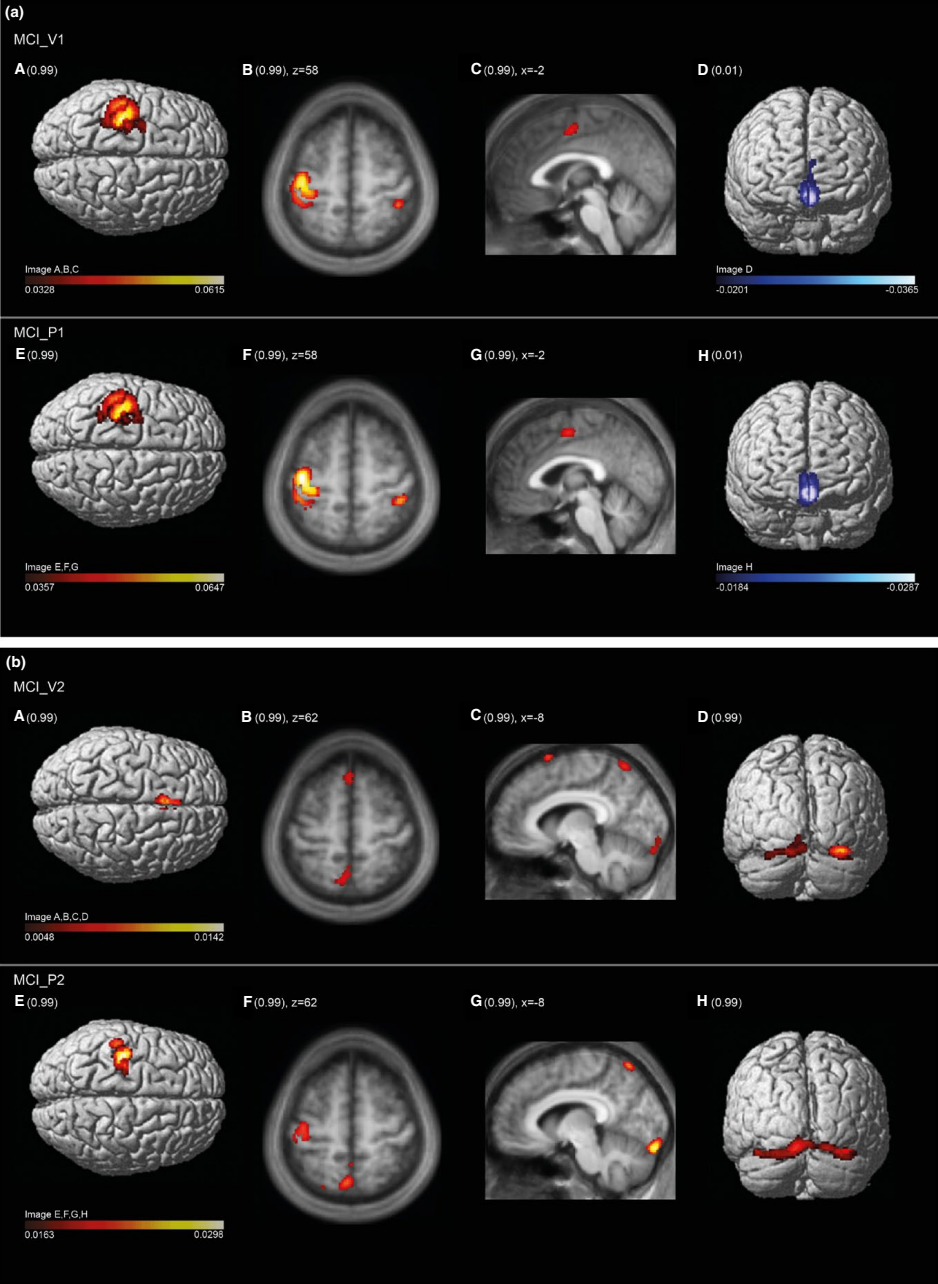
(a) Regions salient in the mean component image MCI_V1 (upper row) and MCI_P1 (lower row). Suprathreshold voxels at 99th percentile and first percentile are overlaid on a surface reconstructed canonical T1 brain image (related to SPM12 (http://www.fil.ion.ucl.ac.uk/spm/software/spm12/)) or a sagittal and axial slice of a mean T1‐image (related to the study cohort). The range of the 99th (dark red to white colorbar) and first quantile (dark blue to white color bar) is indicated. The values relate to the voxel loads, reflecting the voxels’ contribution to a given PC. Images are spatially normalized to MNI space. Note the identical oversight or slice in comparison with the two condition images. There is almost identical involvement of sensorimotor cortices contralateral to moving hand (A, B, E, F), bilateral sensory cortex (B, F), paralimbic anterior cingulate cortex (C, G), and medial fronto‐polar cortex (D, H) at 99th and first quantile, respectively. (b) Regions salient in the mean component image MCI_V2 (upper row) and MCI_P2 (lower row). Suprathreshold voxels at 99th percentile are overlaid on brain structure similarly as described in the legend of Figure 3a. Identical cortical oversights and slices of the two condition images facilitate their comparison. The range of voxel values of the 99th quantile is indicated and associated with the according colorbar. Images are spatially normalized to MNI space. There is common involvement of areas to both MCI_V2 and MCI_P2 condition images, that is, bilaterally at dorsal anterior precuneus (B, C, F, G) and visual cortices (Areas hOc1, hOc2, Oc3v, and hOc4v) (D, H). There are also dissimilarities: the diminished involvement of sensorimotor cortices in comparison with MCI_V1 and MCI_P1 (Figure 3a) varies according to degree, is in MCI_P2 moderately whereas in MCI_V2 severely and subthreshold (E, F). The subthreshold response in sensorimotor cortex in MCI_V2 is accompanied by emergence of activation in the posterior medial frontal cortex; this may suggest a response conflict with respect to the task performance (see main text)

Figure 3a represents the brain regions salient in the mean component image MCI_V1 (upper row) and MCI_P1 (lower row). As reflected by the high vcc of 0.92, the areas expressed in the 99th percentile and the first percentile are almost identical. These include in the 99th percentile the sensorimotor cortices shown in the 3‐D renderings of the cortical surface (A, E), in the transaxial slices (B, F) showing the postcentral gyrus ipsilateral to the moving hand and in the sagittal slices (C, G) showing the paralimbic anterior cingulate cortex. Areas of the first percentile include bilateral projections onto the medial fronto‐polar cortex (D, H). Tables 3a and S3 in Supplemental Material present further details.

**Table 3:** Mean Condition (a) Image 1 of verum group (MCI_V1) (b) Image 2 of placebo group (MCI_P2)

Cluster	Size (n voxels)	MNI (max.)	Anatomical area	Cytoarchitectonic atlas	Functional correlate (see text)
(a)					
99th‐percentile voxels (extension threshold: 32 voxels)					
1+	1330	−34/−26/68	L Precentral g., premotor c.	Area 4a,4p,	I° motor and dorsal premotor cortex SMA
		−28/ −6/66	L Superior frontal g.		
		−50/−30/52	L Postcentral g.	Area 3b,1,2	I° sensory area
2+	77	0/ −4/50	L Dorsal ACC (BA 32)	n.a.	Paralimbic ACC, willed control of action
3+	73	42/−38/ 58	R Postcentral g.	Area 3b,1,2	I° sensory area
First‐percentile voxels (extension threshold: 32 voxels)					
1‐	707	−2/ 54/−12	L Middle orbital g.	Area Fp2 (FPm)	Monitoring action outcomes and Motivation
		2/ 54/−10	R Middle orbital g.	Area Fp2 (FPm)	
		−4/ 62/ 18	L Superior medial g.	Area Fp1 (FPl)	Executive mechanisms
2−	343	−38/−74/44	L Angular g.	Area PGa (IPL)	Heteromodal sensory association cortex
3−	326	50/−66/36	R Angular g.	Area PGp (IPL)	Heteromodal sensory association cortex
4−	76	0/−56/26	L Ventral precuneus	n.a.	Neuronal node of DMN
5−	40	−6/ 60/34	L Superior medial g.	Area Fp1 (FPl)	Executive mechanisms
(b)					
99^th^‐percentile voxels (extension threshold: 32 voxels)					
1+	494	−32/−24/ 70	L Precentral g.	Area 4a	I° motor cortex
		−36/−24/ 68	L Precentral g.	Area 4a,4p	I° motor cortex
		−50/−30/ 52	L Postcentral g.	Area 3b,1,2	I° sensory cortex
2+	710	−10/−92/−10	L,R Striate, parastriate visual c.	Area hOc1, hOc2	I and II° visual cortex
		20/−94/−18	L,R Lingual gyrus	Area hOc3v, hOc4v	III° visual cortex
3+	244	−4/−64/−62	L Medial dorsal anterior precuneus	n.a.	Goal‐directed attention processes
		0/−52/ 66	L Superior parietal lobule	Area 5M	Orienting, shift of attention
4+	41	−26/−70/ 54	L Superior parietal lobule	Area 7A	Orienting, shift of attention
First‐percentile voxels (extension threshold: 32 voxels)					
1−	660	20/−92/ 30	R Superior occipital g.	Area hOc4d, hOc3d	Part of a semantic processing system
		44/−84/ 16	R Middle occipital g.	Area hOc4lp, hOc4la	
			R IPL	Area PGp	Heteromodal sens. association cortex
2−	140	−34/−92/ 18	L Middle occipital g.	Area hOc4lp, hOc4d	See above
			L IPL	Area PGp	See above
3−	500	2/ 34/ −2	R Perigenual and ventral ACC	Area s32, s24	Anticipation of task attention and motivation
		4/ 24/−22	(BA 33, 24, 25)		
4−	41	68/ −26/ 2	R Superior temporal gyrus	Area T3	I° auditory cortex

MNI, coordinates (x,y,z) according to Montreal Neurological Institute space; Cytoarchitectonic atlas, reference to Jülich atlas (Eickhoff et al., 2007); n.a., not applicable; c., cortex; g., gyrus; ACC, anterior cingulate cortex; FPm, fronto‐polar medial; FPl, fronto‐polar lateral; IPL, inferior parietal lobule; DMN, default mode network.

Figure 3b represents the brain regions salient in the mean component image MCI_V2 (upper row) and MCI_P2 (lower row). The moderate vcc of 0.65 between the two image volumes, while highly significant, suggests a more variable pattern than that shown in Figure 3a. Areas in the 99th percentile common to both MCI_V2 and MCI_P2 are bilaterally the dorsal anterior precuneus (B, C, F, G) and visual cortical areas (Areas hOc1, hOc2s, Oc3v, and hOc4v) (D, H). These regions indicate additional visual information processing and, thus, deviation from a pure sensorimotor pattern. Concurrently, involvement of the sensorimotor cortex is diminished in MCI_P2 (E, F) compared to MCI_V1 and MCI_P1 and is subthreshold in MCI_V2. The diminished involvement in MCI_V2 is accompanied by emergence of the posterior medial frontal cortex; this may suggest response conflict interfering with motor activity (Ridderinkhof, Ullsperger, Crone, & Nieuwenhuis, 2004). Tables 3b and S4 in Supplemental Material present further details.

In contrast to the other mean component images that of MCI_V2 could not be reliably assessed due to the small number of contributing subject PCs and their weaker correlations with MCI_K2 (see Table 2). The smaller amplitude in the frequency spectra shown in Figure 1, reflecting smaller eccs and greater fluctuations in their distribution, supports this conclusion.

**Figure 4:**
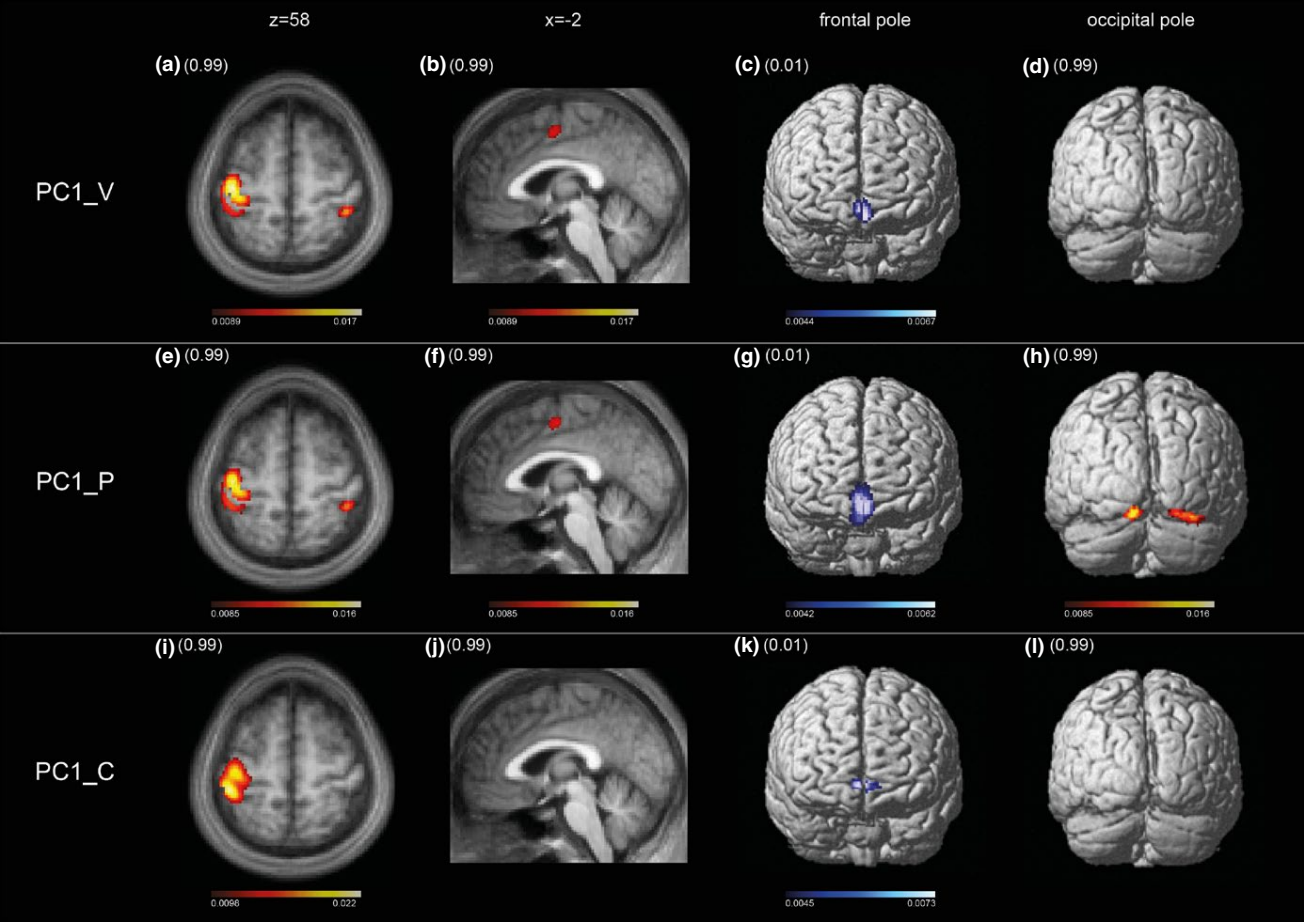
Second‐level PCA as performed to preclude classification bias (see text): regions salient in the first PC image of verum condition PV1_V (upper row), placebo condition PC1_P (middle row), and an age‐matched control group PC1_C (lower row). Suprathreshold voxels at 99th percentile and first percentile are overlaid on brain structure similarly as described in the legend of Figure 3a. Identical cortical oversights and slices of the four condition images facilitate the comparison with mean condition images 1 in Figure 3a (B, C, D, F, G, H) and 2 in Figure 3b (D, H). The range of voxel values of the 99th and first quantile are indicated and associated with the according colorbars. Images are spatially normalized to MNI space. PC1_V and PC1_P have almost identical involvement at sensorimotor cortices contralateral to moving hand, bilaterally at primary sensory cortex and paralimbic anterior cingulate cortex (thresholded at 99th percentile), and medial fronto‐polar cortex (first percentile). PC1_V reflects a pure motor control pattern, whereas PC1_P has an admixture of secondary visual cortex (hOc2 on both sides and hOc3 right), similar to the pattern of MCI_V2 and MCI_P2 (Figure 3a). PC1_C pattern is purely motor comparable to PC1_V, however without involvement of paralimbic anterior cingulate cortex most likely due to rehearsal of the task

The second‐level PCAs of the 41 salient components of verum and 42 of placebo confirm the regular expression of mean component images MCI_V1 and MCI_P1 (Figure 4). Of the 41 principal components of the verum PCA, the first (PC1_V) explained 20.0% of the variance. The dominant pattern of motor control represented by MCI_V1 was replicated almost completely by PC1_V according to the volume correlation of *r *= .97 (*p *< .0000). Of the 42 principal components of the placebo PCA, the first (PC1_P) explained 22.9% of the variance. The dominant pattern of motor control represented by MCI_P1 was well replicated according to the volume correlation of *r *= .89 (*p *< .0000). In contrast to the pattern of PC1_V1 that of MCI_P1 is not purely motor as it includes bilaterally secondary visual cortices, which are also evident in MCI_P2. The difference in volume correlations of the two principal components was significant according to the Fisher transformation test, which yielded z = 19.35 corresponding to *p *< .0000. Thus, PC1_V is a clearer expression of motor control.

Of the 840 principal components derived from the first‐level PCA of the age‐matched control group, 19 task‐related components were subjected to a second‐level PCA. The first PC (PC1_C) explained 22.4% of variance. The volume correlations with both MCI_V1 and MCI_P1 were highly significant, that is, *r *= .65 and *r *= .66, respectively, corresponding in both cases to *p *< .000. Most importantly, the extent of sensorimotor cortex involvement was not different. The slight difference in pattern shown in Figure 4, especially the absence of the paralimbic ACC, is most likely due to rehearsal before scanning (Paus 2001). In contrast to the original cohort, the age‐matched controls performed the motor task immediately after an external cue, that is observation of the task in the scanner.

### 3.4 Dynamic causal modeling

**Figure 5:**
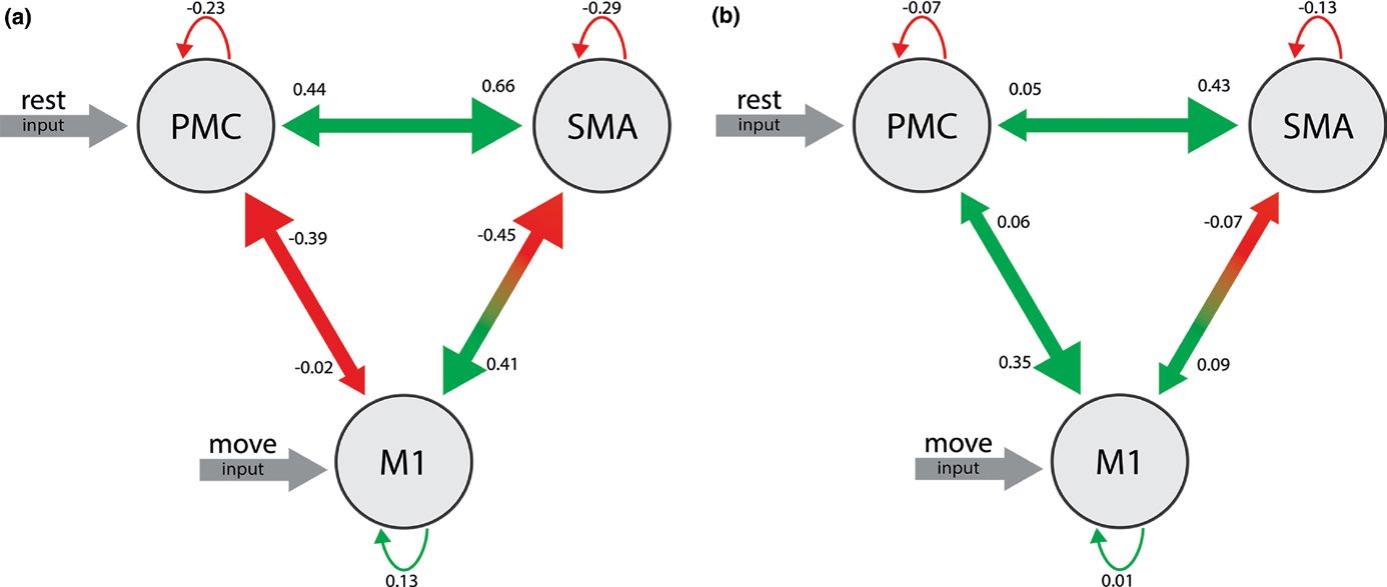
Representation of the best‐fitting dynamic causal model of the three core regions: M1, PMC, and SMA with the inputs “rest” on the PMC and “move” on the M1. Notable is the prominent change after a single dose of escitalopram; the connectivity between M1 and PMC increases in both directions, whereas the self‐related connectivity of PMC and M1 decreases

Of the 38 valid acquisitions, 32 showed significant BOLD activation in the defined motor ROIs according to the selected threshold: *p *< .05, corrected for FWE. The random effects Bayesian algorithm determined the best‐fitting model to be the one with input “rest” on the PMC and “move” on the M1 (Figure 5). Except for the connection between the SMA and itself, all connections yielded coupling parameters differing significantly between placebo and verum conditions according to the Mann–Whitney U test. Among the coupling parameters, a change in sign emerged only in the connection between M1 and PMC. The placebo condition produced coupling parameters between M1 and PMC were −0.39 ± 0.08 and between PMC and M1 −0.02 ± 0.11, whereas the verum condition resulted in 0.06 ± 0.47 and 0.35 ± 0.48 for the same connections, respectively. In both cases, the difference is significant with *p *< .022. Mean coupling parameters and test statistics are shown in Table S5.

## 4 DISCUSSION

In a previous paper, we distinguished two cerebral patterns occurring during a motor fMRI task consisting of manipulation of rectangular parallelepipeds with the fingers (Kagi et al., 2010). The first, cerebral pattern I, appears regularly, reflecting most likely motor control, as a result of concerted, directed, and adaptive motion of the fingers while manipulating and exploring the objects. The second, cerebral pattern II, is an irregularly appearing pattern suggesting motor performance with less task attention and volitional effort. The patterns emerged in principal component analyses (PCA) of seven subjects; task‐related components were identified via the correlation of the hemodynamic response with the component expression coefficients (ecc) and classified according to volume correlations (vccs) among the selected component image volumes, that is, according to their regional similarity. Using the cerebral image volumes of the earlier study as reference, we confirmed the two motor patterns observed in that study regarding both frequency of occurrence and involvement of brain regions. In almost all valid activation runs either cerebral pattern I or II could be discerned.

Based on this categorization, we were able to evaluate our working hypothesis of an effect of escitalopram on the expression of sensorimotor networks by direct comparison of the verum and placebo conditions. First, while finger movement frequency was not different in the two conditions and, thus, the implicated brain regions were comparable in both conditions (Pool, Rehme, Fink, Eickhoff, & Grefkes, 2013), the average ecc of the verum condition associated with cerebral pattern I of motor control was significantly greater than that of the placebo condition. Second, the placebo condition expressed the cerebral pattern II of less controlled motor behavior more frequently than the verum condition while the average ecc showed greater expression in the placebo condition at the trend level. A joint analysis of eccs and vccs using the Mahalanobis metric confirmed in the expression of pattern II a significant difference between the two conditions.

Both the greater expression of cerebral pattern I and the lesser manifestation of cerebral pattern II are indirect signs of a more efficient output of motor control in the verum condition. Or, the relatively high frequency of pattern II in subjects administered a placebo and the anticorrelation of the two patterns suggests that in this condition the subjects had to expend more effort to gain and sustain motor control. Therefore, the administration of escitalopram appears to benefit directed motor control.

The strong anticorrelation between the cerebral activation patterns I and II is a new finding in this study. We conjecture that this anticorrelation indicates the involvement of the default mode network in pattern II as task attention and volitional effort wane. This effect is possibly due to the emergence of task‐independent thoughts, for example reorientation or mind wandering. Mind wandering is a common phenomenon in routine tasks, such as the simple motor hand task of our study, and involves typically the precuneus (Wu, Chan, & Hallett, 2008), a key structure in cerebral pattern II. The anticorrelation between the two cerebral patterns is more pronounced in the new study than in the earlier (Kagi et al., 2010), in which the subject cohort was younger. These effects are manifest in the reduced thumb movement frequency of the older cohort. An age effect as reflected by the extent of involved sensorimotor cortex could not be found in the age‐matched control group.

### 4.1 Cerebral pattern I

The regions of cerebral pattern I delineated by voxels in the highest percentile involve extensively the primary motor and sensory cortex as well as the dorsal premotor cortex and correspond typically to the sensory‐guided motor activity of grasping. This pattern has been attributed to a dorsal–dorsal sensorimotor substream by Binkofski and Buxbaum (Binkofski & Buxbaum, 2013). Effective grasping and object manipulation relate to three properties of the motor system: the capacity to generate independent finger movements, the ability to transform sensory information about the object to be grasped into an appropriate hand configuration, and a somatosensory control of finger movements (Binkofski et al., 1999; Jeannerod, Arbib, Rizzolatti, & Sakata, 1995). Accordingly, the high‐level sensory feedback from muscle spindles and joint receptors during object manipulation might be expressed by the bilateral involvement of the primary sensory cortex, a factor enhancing BOLD response in the primary motor area contralateral to the moving hand (Pool et al., 2013); especially, the implication of paralimbic ACC implies elevated motor control. On the one hand, this structure is functionally interconnected with the anterior ventral compartment of the dorsal lateral prefrontal cortex (dlPFC), a node of the cortico–striato–thalamic loop involved in control of action execution (Alexander, DeLong, & Strick, 1986; Cieslik et al., 2013; DeLong, Alexander, Mitchell, & Richardson, 1986; Haber & McFarland, 2001). On the other hand, the paralimbic ACC receives direct input from medial dorsal thalamus (Eckert et al., 2012), a route subserving focused attention and motivation (Ongur & Price, 2000).

The regions of cerebral pattern I delineated by voxels in the lowest percentile involve several areas of the default mode network, encompassing the ventral medial prefrontal cortex and inferior parietal lobule on both sides and a basal compartment of the left ventral precuneus. Due to its open orientation, the precuneus plays a prominent role in the novel stage of learning before motor programs attain the level of routine (Gusnard, Raichle, & Raichle, 2001; Wu et al., 2008). The appearance in the lowest percentile of this compartment of the precuneus adjacent to the posterior cingulate cortex may indicate the absence of self‐related processing while focusing attention on task performance (Cavanna & Trimble, 2006).

An additional region of pattern I is the frontal pole. Relying on cytoarchitectonically defined maps Bludau et al. (2014) were able to discern two areas within the frontal pole, a lateral and a medial subarea (FPl and Fpm). Coordinate‐based meta‐analysis of functional imaging studies showed both common and separate coactivation of these areas. Both FPl and FPm subareas are part of the human ventral lateral frontal cortex, that is, human vlFC region, as established by Neubert, Mars, Thomas, Sallet, & Rushworth (2014) using structural and functional neuroimaging methods. Derived from resting‐state fMRI functional connectivity patterns, their study delineated distinct neural networks related to FPl and FPm. The former was shown to be connected with dlPFC and angular gyrus (PGa, PGp), the latter with temporal pole, posterior cingulate cortex, and amygdala. The FPl is unique in humans having no counterpart, for example, in the macaque prefrontal brain region. The function of this subarea appears to be the execution of specific higher order attention (Kouneiher, Charron, & Koechlin, 2009; Orr, Smolker, & Banich, 2015) in contrast to the attention to simple task performance maintained by, for example, the dlPFC. Concerning Fpm, metadata based on the BrainMap database suggests that it is mainly involved in higher order emotional processing (http://www.brainmap.org, Fox & Lancaster, 2002).

The remaining regions found in cerebral pattern I are the posterior temporal and inferior frontal areas, middle frontal cortex, and the angular gyrus subareas, PGa and Pgp. Connectivity and coactivation patterns suggest that these areas may play a significant role in the ventral attention network (Vossel, Geng, & Fink, 2014).

### 4.2 Cerebral pattern II

Remarkable in the highest percentile of cerebral pattern II is the significantly smaller average area of the sensorimotor cortex compared to the corresponding area in pattern I, a further aspect of diminished motor output in this condition. In particular, the extent of the dorsal premotor cortices is considerably diminished and there is no involvement of primary sensory cortex ipsilateral to the moving hand. In addition to this area, the pattern captures areas of multiple sensory afferences, including visual and auditory areas. A prominent feature of the pattern is the involvement of precuneus, similarly observed in our earlier study (Kagi et al., 2010). As in that study, the ventral precuneus appears among the voxels in the lowest percentile of cerebral pattern I, while its upper and anterior compartments appear among the voxels in the highest percentile of pattern II, where they are associated with the motor cortex (Margulies et al., 2009). It has been suggested that functional connections between precuneus and motor areas change in relation to the degree of motor control (Wu et al., 2008). Accordingly, the precuneus attains more effective connectivity during diminished motor control, for example, during learning of hand motor skills or diminished attention to task performance (Culham, Cavanagh, & Kanwisher, 2001; Sakai et al., 1998). Interpretation of pattern II as manifestation of dual‐task performance is supported by the appearance of visual association areas and the superior parietal lobule among the voxels in the highest percentile of the pattern. The involvement of the ventral visual path indicates that areas engaged in object, color and shape recognition are part of the pattern not related to the primary goal of the task (Caspers et al., 2013; Eickhoff, Rottschy, Kujovic, Palomero‐Gallagher, & Zilles, 2008). And the superior parietal lobule is a region implicated when subjects shift their attention between any two types of input, for example, between two different sensory modalities (Behrmann, Geng, & Shomstein, 2004). Finally, dual‐task performance is a characteristics of the function of the precuneus (Sakai et al., 1998).

Regions with voxels in the lowest percentile of cerebral pattern II include the dorsal visual stream (Eickhoff et al., 2008). The related dorsal visual association cortices and the angular gyrus, involved on both sides, are associated functionally with mindful attention. However, regions in the lowest percentile of this pattern covary in opposition to the regions in the highest percentile involved in task performance. Thus, their appearance and that of the subgenual and ventral ACC may rather indicate a state of immersion in mind wandering (Lebois et al., 2015).

### 4.3 Second‐level PCA as control for biases in volume correlation analysis

Second‐level PCAs of task‐related first‐level PCs for each condition confirmed the main findings of the volume correlation analysis: The motor control pattern expressed in the first PC was essentially identical in the verum condition and highly similar in the placebo condition. According to the Fisher transformation test, the difference in expression is highly significant and the verum condition expressed the pattern more strongly. Furthermore, the motor control pattern in the placebo condition is qualitatively altered by emergence of the visual association cortices (hOc2s, Oc3v), the same regions observed in cerebral pattern II in both the verum and placebo conditions. This corroborates the hypothesis mentioned above that the placebo condition was prone to interference of mental processing with motor control. Controlling specifically for thumb frequency in age‐matched subjects showed a similar motor control pattern and verified equal involvement of sensorimotor cortex contralateral to the moving hand.

### 4.4 Effect of escitalopram

From a neurophysiological point of view, the motor effect of escitalopram observed represents most likely an early phase of sensorimotor facilitation persisting a few hours, mediated by glutamate interaction with sensitized NON‐NMDA receptors in the postsynapse as described in the introduction (Kandel, 2000). Jacobs et al. (2002) characterized the structure and function of the serotonin system within the brain on the basis of animal experiments in cats. Most importantly, they described a consistent increase in motor output upon application of serotonin. In particular, serotonin facilitates not only tonic motor response, but also generates phasic activity during repetitive movements such as locomotion, respiration, and chewing. Jacobs et al. (Jacobs & Fornal, 1997) recognized also a link to the sleep/wake cycle and accordingly a general coactivation of the serotonin system with enhanced alertness and activity. Furthermore, sensory information decreases concurrently with enhanced motor output after application of a serotonergic agent. And reciprocally, motor function is hindered by brief inactivation of the serotonin system, for example during orientation to salient stimuli, when sensory information processing is facilitated (Jacobs & Fornal, 1995). Recent findings of Klaassens et al. (2015) confirm these observations. Exploring whole‐brain functional connectivity in the resting state after application of sertraline, a serotonergic agent, they discovered a decreasing functional connectivity between precuneus and the sensorimotor network. Dorsal anterior precuneus, emergent during the placebo condition in this study, is distinguished by its important role in orientation and attention as could be shown also in activation studies using functional imaging of visuo‐spatial imagery (Cavanna & Trimble, 2006; Wenderoth, Debaere, Sunaert, & Swinnen, 2005). Loubinoux et al. (2002) could verify an enhanced motor output in normal volunteers after a single dose of paroxetine, a relatively specific serotonin reuptake inhibitor, using paced complex finger sequence movements. The increased response involved the primary motor cortex and posterior part of supplementary motor area. In patients with a pure motor hemiparesis due to lacunar infarct of pyramidal tract, Pariente et al. (2001) found an increased activation within the primary motor hand area using fMRI and paced finger flexions of the affected hand. The improved motor hand function corroborated this neuroimaging finding.

As assessed by fMRI of not‐paced, phasic exploratory finger movements grasping rectangular cuboids, we established an enhanced motor output after application of escitalopram. Novel in our findings is a differentiated view of the involved motor networks: 1. The serotonergic agent supported the expression of a motor control network in contrast to a less voluntarily motor network more prominent in the placebo condition. Applied to the motor core area of this distributed neuronal network, the effective connectivity explored with dynamic causal modeling confirms the enhanced motor output resulting from significantly increased connectivity between M1 and PMC, characteristic of a sensory‐guided motor skill. 2. During diminished voluntary motor control, the precuneus emerges as a key area of a second motor network associated with a functional increase in areas of sensory modalities unrelated to the task, for example the superior parietal lobule and secondary visual association areas. The most likely source of this increase may be mental activities, for example re‐orienting or mind wandering. The interrelationship of motor output and reorientation as expressed by the interaction between the motor cortex and precuneus corroborates in humans the proposal of Jacobs et al. concerning the serotonergic system (Jacobs & Fornal, 1997).

### 4.5 Limitations

The pilot study reported here enlisted a cohort of ten healthy volunteers suited for a study of poststroke pharmacotherapy. Although a small number, this cohort size permitted validation of cerebral pattern I and established a difference in expression between verum and placebo conditions with convincing statistical significance. However, validation and comparison of cerebral pattern II, dominated by the prominent expression of pattern II in the placebo condition, is hindered by the variability associated with the proposed interference of task‐independent cognitive processes. The uncertainty is particularly evident regarding the mean component of the verum condition which is composed of only five PCs. This is reflected in the frequency power spectrum of the associated mean temporal expression coefficient, which exhibits 50% less amplitude of the main peak than the other mean components, as shown in Figure 1. A larger cohort would permit a more reliable comparison between placebo and verum conditions as well as analysis of the few task‐related PCs not attributable to one of the patterns. Furthermore, a larger cohort would provide us with the opportunity to address the significance of genotypes possibly involved in treatment response.

The task of this study represents an everyday motor skill easily performed by normal volunteers. In patients recovering from hand paresis, the effort exerted during the task might also implicate the sensorimotor areas, introducing a confounding factor to be determined by precise measurements of associated behavioral parameters (Borg, 1982; Mochizuki et al., 2009).

## 5 CONCLUSION

Serving as a pilot study for poststroke pharmacotherapy, we report here the effect of a single dose of escitalopram on motor task performance in normal volunteers. Principal component analysis of a well‐studied tactile manipulation task investigated using fMRI established the reproducibility of the two networks of sensorimotor activity proposed in our previous study as well as an effect of escitalopram on the expression of the patterns. The dominant pattern of the PCA shows that a single dose of escitalopram enhances motor output in a tactile manipulation task by activating a sensorimotor network characteristic of motor control. A second pattern, predominantly expressed in the placebo condition, reveals an alternative network showing decreased involvement of primary sensorimotor and dorsal premotor cortices and increased involvement of areas associated with cognitive processes. Thus, the effect of a single‐dose application of escitalopram on motor task performance appears to consist of a transient stabilization of motor task performance and diminished interference from cognitive processes.

## CONFLICT OF INTEREST

The authors declare that they have no conflict of interests.
