# Supplementary material for: The effect of a single dose of escitalopram on sensorimotor networks
Source: Brain Behav. 2018 Apr 20;8(6):e00975. doi: 10.1002/brb3.975 (PMC5991571; doi:10.1002/brb3.975)
Supplement: Supplementary file 1 [file BRB3-8-e00975-s001.pdf]

ONLINE SUPPLEMENTARY MATERIAL

The effect of a single dose of escitalopram on sensorimotor networks

Christian Weisstanner[1], Georg Kägi[2], Werner Krammer[1,2], Chin B. Eap[3], Roland Wiest[1], John H. Missimer[4]**, Bruno J. Weder[1]**

1 Support Center for Advanced Imaging (SCAN), Department of Diagnostic and Interventional Neuroradiology, Inselspital, Bern University Hospital, Bern, Switzerland

2 Department of Neurology, Kantonsspital St. Gallen, St. Gallen, Switzerland.

3 Département de psychiatrie, Unité de biochimie et psychopharmacologie Clinique, CHUV, Lausanne

4 Paul Scherrer Institute, PSI, Laboratory of Biomolecular Research, Villigen, Switzerland.

Index

		Page
Figure S1.	Comparison mean component temporal expression coefficients with the modelled hemodynamic response	2
Figure S2.	Joint distributions of eccs and vccs in placebo and verum conditions	3
Table S1.	Examination of the age-matched control group	4
Table S2.	Coupling constants DCM	5
Table S3.	Functional regions of MCI_P1	6
Table S4	Functional regions of MCI_V2	7
Table S5	ROI coordinates DCM (center of gravity)	8

Figure S1

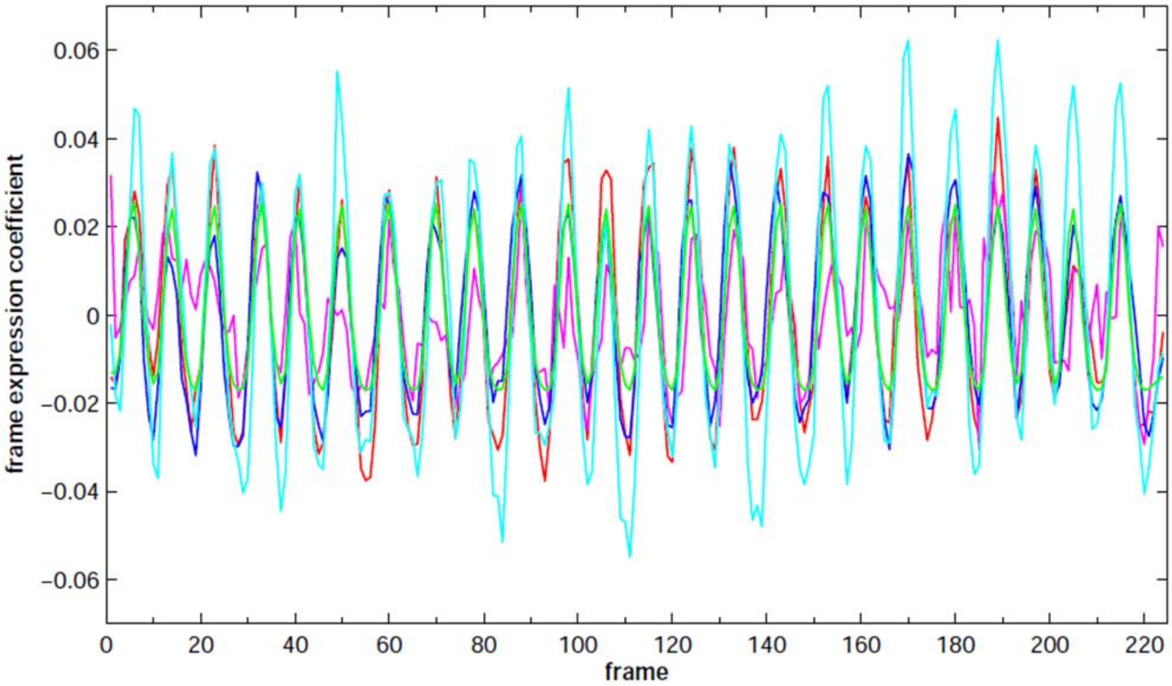

Comparison mean component temporal expression coefficient for MCI_V1 and 2, MCI_P1 and 2 with the modelled hemodynamic response, where red denotes MCI_V1, magenta MCI_V2, blue MCI_P1 and cyan MCI_P2, green the modeled hemodynamic response.

Figure S2

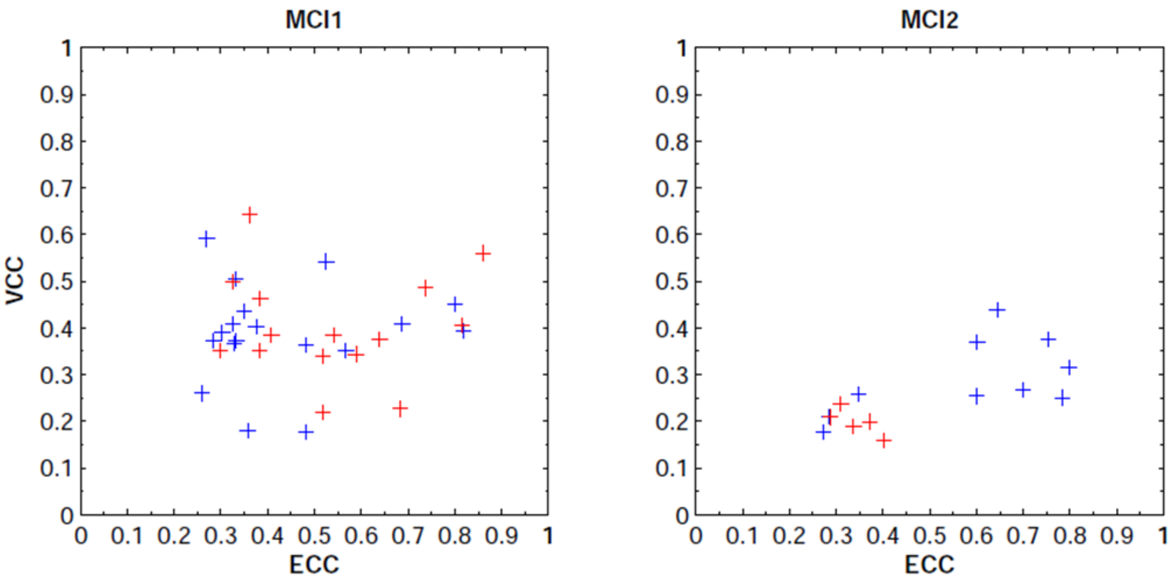

Joint distributions of eccs and vccs in placebo (blue) and verum (red) conditions. On the left the scatter plot of distributions for MCI_V1 and MCI_P1; the correlation coefficients between ecc and vcc are 0.0631 for placebo and -0.0268 for verum. On the right is the scatter plot of distributions for MCI_V2 and MCI_P2; the correlation coefficients between ecc and vcc are 0.5743 ($p < 0.08$) for placebo and -0.7741 ($p < 0.12$) for verum.

Table S1. Examination of the age-matched control group

Image acquisition

Magnetic resonance imaging (MRI) was performed in a 3 T MAGNETOM PRISMA MRI sanner (Siemens, Germany). The protocol included acquisition of a high-resolution T1-weighted Modified Driven Equilibrium Fourier Transform (MDEFT) sequence (TR 7.93 ms, TE 2.49 ms, FA 16°, 176 slices with slice thickness 1 mm; field of view 256 mm with matrix size 256 × 256) for anatomical coregistration. An Echo Planar Imaging (EPI)-BOLD sequence (TR 3000ms, TE 30 ms, FA 90°, 49 transaxial slices with slice thickness 2 mm, field of view 192 mm with matrix size 94 x 94) was used to acquire 144 whole brain slices during the manipulation task.

fMRI blockdesign-task of the age-matched control group

The fMRI session of the age-matched control group consisted of three conditions while the subjects were observing a video-screen: visual fixation on a stationary image showing a cube in one hand while being relaxed (representing baseline), passive viewing of the manipulation task performed with a thumb frequency of 1 Hz, and performance of the task as observed during viewing while fixating on the stationary image again. Each condition was initiated by a visual cue. Each visual cue of 4 seconds was followed by the according condition of 20 seconds. This whole block was repeated 6 times without break leading to a task duration of 7.2 minutes.

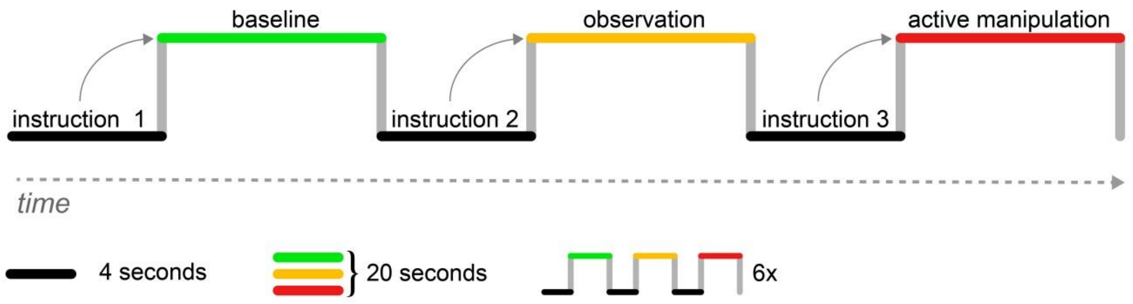

For fMRI analysis only time frames for the conditions „active manipulation“ minus „baseline (i.e. visual fixation, see above)“ were analysed of total 84 acquisitions.

Analysis of finger movements

Thumb frequencies of each active exploration sequence were analyzed using close-up video recorded with a MRI-suitable camera system (12M camera, MRC Systems, Heidelberg, Germany). The thumb frequency was 1.02 ± 0.09 Hz (mean ± SD) for the 10 volunteers in the age-matched control group.

Table S2. Coupling constants DCM

ROI connections	placebo (N=15)		Escitalopram (N=17)		Mann-Whitney-U-Test
	mean (±SD)	variance	mean (±SD)	variance	
PMC → PMC	-0.23 (±0.03)	0.001	-0.07 (±0.15)	0,021	p = 0.003
M1 → M1	0.13 (±0.08)	0,007	0.01 (±0.16)	0,026	p = 0.030
SMA → SMA	-0.29 (±0.13)	0,016	-0.13 (±0.20)	0,041	p = 0.052
PMC → M1	-0.02 (±0.11)	0,012	0.35 (±0.48)	0,226	p = 0.022
PMC → SMA	0.66 (±0.20)	0,039	0.43 (±0.30)	0,091	p = 0.004
M1 → PMC	-0.39 (±0.08)	0,006	0.06 (±0.47)	0,220	p = 0.022
M1 → SMA	-0.45 (±0.24)	0,060	-0.07 (±0.43)	0,186	p = 0.022
SMA → PMC	0.44 (±0.19)	0,037	0.05 (±0.44)	0,195	p = 0.027
SMA → M1	0.41 (±0.21)	0,043	0.09 (±0.38)	0,144	p = 0.020

Table S3. Mean Condition Image 1 of placebo group (MCI_P1)

Cluster	Size (n voxels)	MNI (max.)	Anatomical area	Cytoarchitectonic atlas	Functional Correlate (see text)
99th-percentile voxels (extension threshold: 32 voxels)					
1+	1291	-34/-26/66	L Precentral g., Premotor c.	Area 4a,4p	I° motor and dorsal premotor area
		-30/ -8/66	L Superior frontal g. lat.		SMA
		-50/-30/52	L Postcentral g.	Area 3b,1,2	I° sensory area
2+	78	0/ -4/52	L Dorsal ACC (BA 32)	n.a.	Paralimbic ACC, willed control of action
3+	98	44/-36/58	R Postcentral g.	Area 3b,1,2	I° sensory area
1st-percentile voxels (extension threshold: 32 voxels)					
1-	707	-2/ 54/-12	L Middle orbital g.	Area Fp2 (FPm)	Monitoring action outcomes
		2/ 4 /-10	R Middle orbital g.	Area Fp2 (FPm)	and Motivation
		2/ 42/-16	R Rectal g.	Area Fo1	
2-	267	-36/-80/40	L IPL, Angular g.	Area PGa, PGp	Heteromodal sensory association cortex
			R IPL, Angular g.		
3-	180	54/-66/28		Area PGp, PGp	Heteromodal sensory association cortex
			L Ventral precuneus		
4-	76	0/-56/26	L Temporal pole	n.a.	Neuronal node of DMN
5-	51	-6/ 60/ 34	R, (L) Ventral ACC	n.a.	Neuronal node of DMN
6-	36	2/ 34/ -4		Area s24, 33	Anticipation of task attention and motivation

MNI, coordinates (x,y,z) according to MNI (Montreal Neurological Institute) space; Cytoarchitectonic atlas, reference to Jülich atlas (Eickhoff, et al., 2007); n.a., not applicable; c., cortex; g., gyrus; ACC, anterior cingulate cortex; FPm, Fronto-polar medial; FPL, Fronto-polar lateral; IPL, Infero-parietal lobule; DMN, default mode network

Table S4 Mean Condition Image 2 of verum group (MCI_V2)

Cluster	Size (n voxels)	MNI (max.)	Anatomical area	Cytoarchitectonic atlas	Functional Correlate (see text)
99th-percentile voxels (extension threshold: 32 voxels)					
1+	693	0/-28/ 74	L, (R) SPL	Area 7A, 5M, 5L	Goal-directed attention processes
		-6/-60/ 62	L Precuneus	n.a.	Motor part of precuneus
2+	345	-2/-90/-16	L,R Striate, parastriate visual c.	Area hOc1, hOc2	I and II° visual cortex
		-12/-90/-20	L Lingual gyrus	Area hOc3v hOc4v	III° visual cortex
3+	216	-2/-24/ 70	L Posterior medial frontal cortex	n.a.	Posterior medial frontal c, response conflict
4+	201	32/-80/-26	R Lingual g.	Area hOc3v hOc4v	III° visual cortex
1st-percentile voxels (extension threshold: 32 voxels)					
1-	1227	20/-92/30	R IPL	Area PF, PFm	Somatosensory association cortex
		44/-84/16	R IPL	Area PGp	Transmodal information transfer
		70/-28/ 4	R Superior temporal g.	Area T3	I° auditory cortex
2-	62	54/ 20/12	R Inferior frontal g. Pars opercularis	Area 44,45	Context dependent narrative comprehension
3-	43	0/-18/ -2	R,L Medial-dorsal thalamus	Thal: frontal<br>Thal: temporal	Node of dorsolateral-prefrontal circuit
4-	40	68/-26/ 2	R Hippocamus	Dentate gyrus, CA1	Declarative memory

MNI, coordinates (x,y,z) according to MNI (Montreal Neurological Institute) space; Cytoarchitectonic atlas, reference to Jülich atlas (Eickhoff, et al., 2007); c., cortex; n.a., not applicable; g., gyrus; c., cortex; SPL, Superior parietal lobule; IPL, Infero-parietal lobule

Table S5. ROI coordinates DCM (center of gravity)

	placebo (n=15)						verum (n=17)					
	run 1			run 2			run 1			run 2		
ID	PMC	M1	SMA	PMC	M1	SMA	PMC	M1	SMA	PMC	M1	SMA
1	-38/-10/60	-42/-24/46	-4/-6/52	-32/-8/70	-44/-20/54	0/14/62	-	-	-	-34/-10/64	-40/-26/48	-4/-2/50
2	-44/-16/54	-36/-30/50	-4/-10/54	-40/-22/60	-22/-30/76	0/2/52	-44/-26/68	-32/-36/60	-2/-2/66	-40/-24/64	-36/-30/56	2/-6/58
3	-	-	-	-	-	-	-40/-14/60	-28/-32/64	-4/4/54	-	-	-
4	-36/-14/68	-34/-32/60	-2/6/46	-	-	-	-28/-18/70	-40/-24/56	-8/-2/48	-26/-12/76	-38/-28/76	-8/-10/56
5	-	-	-	-	-	-	-34/-10/66	-32/-28/66	-4/-4/56	-40/-16/64	-38/-32/70	-4/-6/52
6	-42/-22/58	-36/-38/60	-2/18/40	-42/-26/64	-40/-40/62	2/-4/62	-36/-16/68	-36/-30/68	0/18/40	-34/-10/70	-38/-38/60	-2/12/44
7	-22/-2/66	-32/-26/60	0/22/32	-26/-8/68	-28/-32/62	-2/2/46	-30/-14/76	-34/-28/68	-2/0/60	-26/-6/72	-36/-26/62	-2/-2/60
8	-38/-24/66	-44/-38/56	2/-6/54	-40/-22/64	-42/-44/56	-2/-6/56	-38/-22/68	-48/-18/52	-2/-8/58	-42/-24/64	-42/-40/60	0/-14/58
9	-38/-14/64	-40/-24/58	0/-6/56	-36/-18/70	-48/-28/56	-2/-6/54	-40/-16/66	-46/-28/54	-2/-6/56	-38/-16/64	-46/-30/54	0/0/56
10	-34/-20/72	-34/-34/70	-2/-6/54	-44/-14/68	-40/-30/56	-2/-4/56	-	-	-	-42/-18/66	-40/-30/56	-2/-4/56
